# Identification of Cerebrospinal Fluid Metabolites as Biomarkers for Enterovirus Meningitis

**DOI:** 10.3390/ijms20020337

**Published:** 2019-01-15

**Authors:** Dominica Ratuszny, Kurt-Wolfram Sühs, Natalia Novoselova, Maike Kuhn, Volkhard Kaever, Thomas Skripuletz, Frank Pessler, Martin Stangel

**Affiliations:** 1 Clinical Neuroimmunology and Neurochemistry, Department of Neurology, Hannover Medical School, Carl-Neuberg-Str. 1, 30625 Hannover, Germany; Ratuszny.Dominica@mh-hannover.de (D.R.); Suehs.Kurt-Wolfram@mh-hannover.de (K.-W.S.); Skripuletz.Thomas@mh-hannover.de (T.S.); 2 Centre for Individualised Infection Medicine, Feodor-Lynen-Str. 15, 30625 Hannover, Germany; 3 Institute for Experimental Infection Research, TWINCORE Centre for Experimental and Clinical Infection Research GmbH, Feodor-Lynen-Str. 7, 30625 Hannover, Germany; novos65@mail.ru (N.N.); maike.kuhn@t-online.de (M.K.); 4 United Institute of Informatics Problems, the National Academy of Sciences of Belarus, Surganova 6, 220012 Minsk, Belarus; 5 Research Group Biomarkers for Infectious Diseases, Helmholtz-Centre for Infection Research, Inhoffenstr. 7, 38124 Braunschweig, Germany; 6 Research Core Unit Metabolomics, Hannover Medical School, Carl-Neuberg-Str. 1, 30625 Hannover, Germany; kaever.volkhard@mh-hannover.de

**Keywords:** enterovirus, cerebrospinal fluid, meningitis, CNS infection, metabolomics, biomarker, phosphatidylcholines

## Abstract

Enteroviruses are among the most common causes of viral meningitis. Enteroviral meningitis continues to represent diagnostic challenges, as cerebrospinal fluid (CSF) cell numbers (a well validated diagnostic screening tool) may be normal in up to 15% of patients. We aimed to identify potential CSF biomarkers for enteroviral meningitis, particularly for cases with normal CSF cell count. Using targeted liquid chromatography-mass spectrometry, we determined metabolite profiles from patients with enteroviral meningitis (*n =* 10) and subdivided them into those with elevated (*n =* 5) and normal (*n =* 5) CSF leukocyte counts. Non-inflamed CSF samples from patients with Bell’s palsy and normal pressure hydrocephalus (*n =* 19) were used as controls. Analysis of 91 metabolites revealed considerable metabolic reprogramming in the meningitis samples. It identified phosphatidylcholine PC.ae.C36.3, asparagine, and glycine as an accurate (AUC, 0.92) combined classifier for enterovirus meningitis overall, and kynurenine as a perfect biomarker for enteroviral meningitis with an increased CSF cell count (AUC, 1.0). Remarkably, PC.ae.C36.3 alone emerged as a single accurate (AUC, 0.87) biomarker for enteroviral meningitis with normal cell count, and a combined classifier comprising PC.ae.C36.3, PC.ae.C36.5, and PC.ae.C38.5 achieved nearly perfect classification (AUC, 0.99). Taken together, this analysis reveals the potential of CSF metabolites as additional diagnostic tools for enteroviral meningitis, and likely other central nervous system (CNS) infections.

## 1. Introduction

A variety of processes can lead to meningeal inflammation. Viruses are the most common cause of infectious meningitis, followed by bacteria. Fungal or parasite infections are found rarely. There is also a variety of causes of non-infectious meningeal inflammation, such as carcinomatosis, adverse drug effects, or autoimmune diseases. Pathogens can reach the meningeal structures via the blood stream and infiltrate the membranes that surround the brain and spinal cord. Another way of infection is by infiltration *per continuitatem* when circumambient tissue is damaged. Typical symptoms of meningitis are headache, fever, or neck stiffness. Altered mental status or neurological deficits appear additionally when meningitis evolves into meningoencephalitis, in which brain tissue is also affected [1,2].

Of all neurotropic viruses that can cause inflammation of the meninges, enterovirus infections are the most common, comprising 40–77% of all cases. Infections with herpes simplex virus (HSV) or varicella zoster virus (VZV) are less common [3]. Enterovirus species belong to the picornaviridae group, with seven human species: enterovirus A–D, and rhinovirus A–C [4]. A specific treatment is not available and the disease is usually self-limiting without persistent sequelae. Although enterovirus meningitis may cause only non-specific symptoms, such as general malaise and decreased daily activity, a correct diagnosis is important in order to exclude conditions that are more serious or require different treatments. A lumbar puncture (commonly referred to as spinal tap) is the commonly performed procedure to obtain cerebrospinal fluid (CSF) to diagnose meningitis [5,6]. The most rapidly available results from a lumbar puncture include cell count, total protein, and lactate concentrations. In this context, a pleocytosis (increased leukocyte count of ≥5 cells per μL) is indicative of an inflammatory process, regardless of whether it is of infectious or non-infectious origin [7]. Caution is required since CSF cell count is normal in up to 15% of patients with enterovirus meningitis [8]. Thus, if clinical symptoms suggest a diagnosis of meningitis, there is a need for additional diagnostic biomarkers to identify these cases of enteroviral meningitis despite the absence of pleocytosis [9,10]. Except for the detection of enteroviral RNA via PCR, there is no specific marker for this form of meningitis yet [11]. Additionally, turn-around time of the PCR test may be long (several hours if the test can be performed onsite and on the same day, but mostly up to several days because these specialized tests are not run on a daily basis, or if the sample needs to be sent to an external laboratory) and it may not always be available. We have recently shown that alterations in CSF metabolites lead to highly specific biomarkers for distinct forms of VZV reactivation, including for differentiation between the clinically similar facial nerve zoster (VZV reactivation involving the facial nerve) and idiopathic Bell’s palsy [12]. We have therefore analyzed metabolite populations in CSF of patients with enteroviral meningitis, with and without pleocytosis, in order to identify metabolite biomarkers for (1) enteroviral meningitis per se and (2) specifically for the differentiation between enteroviral meningitis with normal CSF cell count and non-inflamed, non-infected control samples. Furthermore, this analysis may afford insight into pathophysiological processes during the infection. 

## 2. Results

### 2.1. Demographic and Standard Blood and CSF Diagnostic Parameters

Sociodemographic and routine laboratory parameters are summarized in Table 1. Consistent with the well-documented natural history of enteroviral meningitis [8], it was characterized by a relatively mild degree of neuroinflammation, as evidenced by a mild-to-moderate pleocytosis in those patients with ≥5 cells/µL, mildly increased lactate concentrations, and mild-to-moderate blood-CSF-barrier (BCB) dysfunction (Table 1). There were no differences in peripheral blood leukocyte counts or CRP values among the four groups. When comparing the enterovirus subgroups with or without pleocytosis against controls, only the IgG index differed significantly between the group without pleocytosis and controls. In contrast, enterovirus patients with pleocytosis (1) were significantly younger than the controls and the enterovirus patients without pleocytosis, (2) had significantly higher lactate concentrations, and (3) more frequent BCB dysfunction than controls. Thus, while the enteroviral meningitis group as a whole reflected findings typical for this mild-moderately severe central nervous system (CNS) infection, the subgroup with normal CSF cell count was nearly indistinguishable from the control group on the basis of routine CSF diagnostic parameters.

### 2.2. Efficiency of CSF Metabolite Detection by Mass Spectrometry

**Figure 1:**
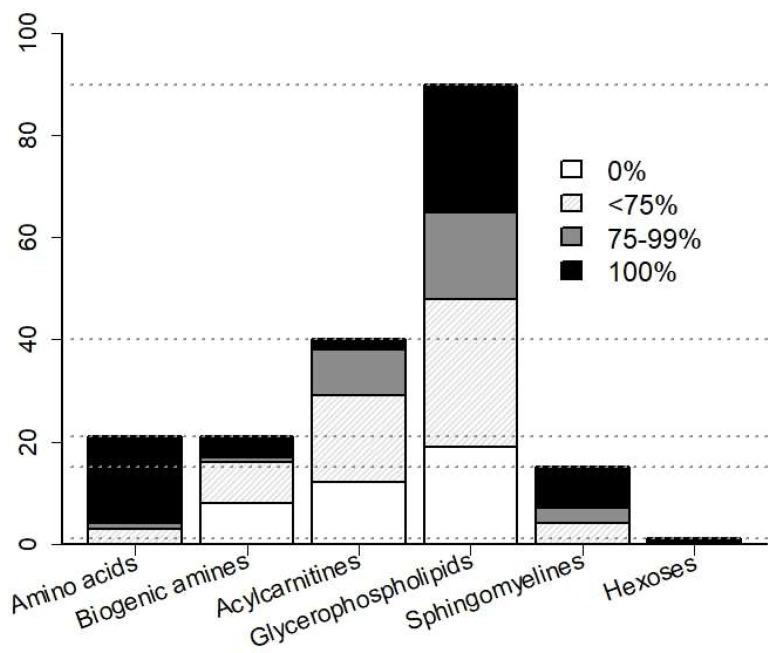
Detection efficiency of metabolites in CSF and selection for subsequent analyses. The number of detectable metabolites per analyte class is stated on the y-axis. Bars indicate the number of analytes in a given class, with concentrations > limit of detection in all samples according to four categories, as indicated by the fill patterns: detected in all samples (“100%”), >75% of samples (“75–99%”), <75% of samples (“<75%”), and in none of the samples (“0%”). The 89 metabolites detected in 75–100% of all samples were used for subsequent analysis.

Of the 188 potentially detectable metabolites, 89 passed quality assessment because of detection >LOD in ≥75% samples (Figure 1). Overall, detection efficiency decreased in the order of amino acids (highest efficiency) > sphingolipids > glycerophospholipids > biogenic amines > acylcarnitines (lowest efficiency). An additional two analytes (kynurenine and acetyl ornithine) were selected from the remaining 99 analytes because of preferential expression in at least one of the enteroviral groups. The resulting 91 analytes comprised 18 amino acids, 7 biogenic amines, 11 acylcarnitines, 43 glycerophospholipids, 11 sphingolipids, and the sum of hexoses. 

### 2.3. Metabolite Reprogramming in CSF in Enteroviral Meningitis

**Figure 2:**
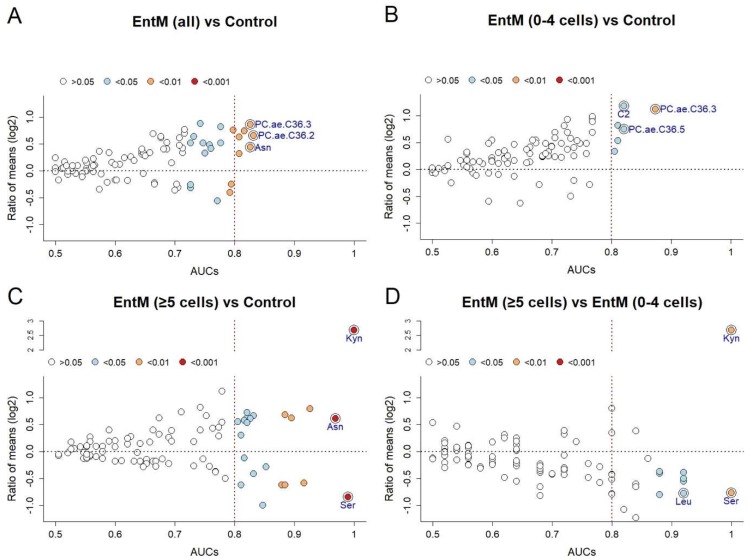
Pronounced alterations in CSF metabolite populations in enteroviral meningitis. (**A**) Enteroviral meningitis (all, *n =* 10) vs. controls (*n =* 19). (**B**) Enteroviral meningitis (0–4 cells/µL CSF, *n =* 5) vs. controls. (**C**) Enteroviral meningitis (≥5 cells/µL CSF, *n =* 5) vs. Controls. (**D**) Enteroviral meningitis (≥5 cells/µL CSF) vs. enteroviral meningitis (0–4 cells/µL CSF). Data are based on comparisons between enteroviral meningitis (all, *n =* 10) or each of the subgroups (normal cell count, elevated cell count, *n =* 5 each) with the non-inflamed control group (*n =* 19). The group with a higher degree of neuroinflammation is listed first, and also formed the enumerator to compute the ratio of mean concentrations and the positive state in ROC analysis. Each circle corresponds to one CSF metabolite. The labels in the graphs identify the 3 metabolite biomarkers with highest AUC. Y-axis: ratio of mean concentrations (“fold change”). X-axis: AUC in binary ROC analysis. Fill color: asymptotic significance of the corresponding ROC curve.

We then used a combination of differential concentration analysis and binary receiver operator characteristic (ROC) curve analysis to assess the degree of concentration changes in the 91 metabolites and to screen the resulting biomarker potential (Figure 2). A marked reprogramming was evident between enteroviral meningitis (all) and controls, as concentrations of 23 metabolites (25%) differed significantly between the two groups. Both increased (*n =* 18) and decreased (*n =* 5) concentrations were detected (Figure 2A). As expected, there also were pronounced differences between enteroviral meningitis with pleocytosis and controls (22 differential concentrations, 24%; 13 increased and 9 decreased; Figure 2C), but even between the non-pleocytosis subgroup and controls, concentrations of 7 metabolites (8%, all increased) changed significantly (Figure 2B). When comparing the pleocytosis vs. the non-pleocytosis subgroups, a tendency toward downregulation in the pleocytosis subgroup was noted, except for a pronounced increase in kynurenine concentration (Figure 2D). This metabolite “reprogramming” in both enteroviral subgroups revealed several promising biomarker candidates among the metabolites. Using a cut-off of AUC > 0.8 for a sufficiently accurate biomarker candidate [13], there were 6 markers for the distinction between enteroviral meningitis (all) and controls (highest three AUC: PC.ae 36.2, asparagine, PC.ae.36.3), 22 for the pleocytosis subgroup vs. controls (highest AUC: kynurenine, serine, and asparagine), 6 for the distinction between the non-pleocytosis subgroup vs. controls (highest AUC: PC.ae.C36.3, C2, PC.ae.C36.5), and 10 (highest AUC: kynurenine, serine, leucine) for the differentiation between pleocytosis vs. non-pleocytosis. Taken together, this analysis revealed a considerable reprogramming of CSF metabolite populations in enteroviral meningitis per se, but also clear differences depending on the presence or absence of pleocytosis. 

### 2.4. Selection of Specific Biomarkers by Internal Cross-Validation

**Figure 3:**
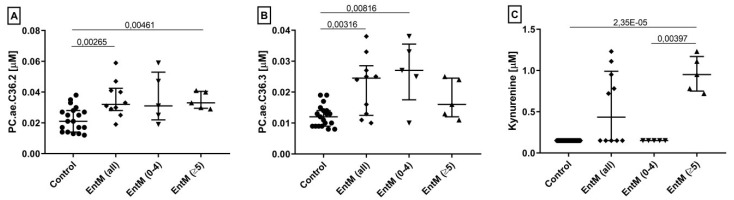
The three best validated CSF metabolite biomarkers for enteroviral meningitis: concentrations in non-inflamed non-infected controls (*n =* 19) and enteroviral meningitis (*n =* 10), or its subgroups with normal (0–4 cells/µL CSF, *n =* 5) or elevated (≥ 5 cells/µL CSF) leukocyte count. (**A**) PC ae C36.2, best marker for enteroviral meningitis (all) vs. controls. (**B**) PC.ae.C36.3, best for enteroviral meningitis (normal cell count) vs. controls. (**C**) Kynurenine (best for enteroviral meningitis (elevated cell count) vs. enteroviral meningitis (normal cell count); all measured concentrations of this biogenic amine were <LOD in controls and EntM (0–4). Brackets indicate significance of difference in median concentrations between the indicated two groups (Mann-Whitney U test). Abbreviations: EntM, enteroviral meningitis; Kyn, kynurenine; PC, phosphatidylcholine.

Cross-validation by the leave-one-out method was then used to identify the most robust biomarkers. For each comparison, the 10 most frequently selected markers and their parameters from ROC analysis are summarized in Table 2. Overall, metabolite signatures reflecting alterations in amino acid metabolism became evident in the comparisons involving the pleocytosis subgroup, whereas alterations in phospholipid metabolism and fatty acid metabolism (acylcarnitines) contributed most to differences between the non-pleocytosis subgroup and controls. Combining the frequency of selection in the cross-validation, AUC from ROC analysis, and asymptotic *p* values of the ROC curves, we then determined the optimal single biomarker for each comparison. Their concentration ranges are shown in Figure 3 and ROC analysis parameters are listed in Table 3. In the comparisons which were defined by absence or presence of pleocytosis, kynurenine was a perfect biomarker (AUC 1.0) for enteroviral meningitis with pleocytosis, suggesting a close association of its increased synthesis with neuroinflammation. Of note, in those instances where the standard diagnostic biomarkers did not provide perfect discrimination (i.e. controls vs. enterovirus meningitis (all) and controls vs. enterovirus meningitis with normal cell count) the discriminatory accuracy of each of these metabolite biomarkers was greater than that of the best standard diagnostic CSF biomarker. In particular, for the clinically important differentiation of enteroviral meningitis with normal CSF cell count from controls, the AUC of PC.ae.C36.3 (0.87) was substantially greater than that of the only significant standard parameter, IgG index (0.81).

### 2.5. Identification of Optimal Metabolite Classifiers by Random Forest Construction

Combining biomarkers into complex classifiers may improve diagnostic accuracy, particularly when intraclass correlations are negative [14]. Random forest construction led to the identification of classifiers for enteroviral meningitis (all) and the non-pleocytosis subgroup that were superior to the single metabolite biomarkers (Table 3). Consistent with the aforementioned amino acid “signature” in enteroviral meningitis (all), two of the three components of the classifier for this group were amino acids (the third feature being PC.ae.C36.3). Of note, the classifier for the distinction of enteroviral meningits with normal cell count from the control group reached nearly perfect discrimination (AUC 0.99). This classifier consisted of PC.ae.C36.3 and two other phosphatidylcholines, underscoring the differential regulation of phosphatidylcholines in this subgroup. A complex classifier was not identified for enteroviral meningitis with pleocytosis because kynurenine alone provided perfect classification, underscoring the close association of increased kynurenine synthesis with this inflamed subgroup. 

## 3. Discussion

Enteroviral meningitis continues to present diagnostic challenges, as up to 15% of patients with this infection may present with normal CSF cell counts and turn-around of PCR diagnostics may be insufficient (see Introduction). We therefore performed a targeted metabolomics screen of CSF from patients with enteroviral meningitis and identified accurate biomarkers, not only for enteroviral meningitis with the expected elevated CSF leukocyte count, but, notably, also for a subgroup of patients with meningitis and CSF cell counts in the normal range.

Our results showed an elevation of several metabolites in both groups of enteroviral meningitis patients, those with and without pleocytosis. The lack of correlation with an increased cell count in CSF suggests that the elevation of those metabolites is based not only on the inflammatory reaction and modulation of the immune system, but also on specific pathological processes within the meninges. Compared to the standard diagnostic CSF parameters such as leukocyte count, protein, or lactate concentrations, the most robust metabolite biomarkers were more sensitive and specific in diagnosing enteroviral meningitis. Furthermore, selecting complex classifiers comprising three metabolites improved classification accuracy further. These results solidify our previous findings that “metabolite signatures” constitute highly accurate diagnostic biomarkers for CNS infections that may be superior to routine CSF diagnostics [12]. 

Phosphatidylcholines constituted two of the metabolites that were best validated as differentially upregulated in enterovirus meningitis. Of these, PC.ae.C36.2 was most closely associated with enteroviral meningitis independent of pleocytosis, whereas the nearly identical metabolite PC.ae.C36.3 was a highly accurate biomarker for the clinically challenging subgroup of meningitis without pleocytosis. 

Phosphatidylcholines are important constituents of the cell membranes in eukaryotes. They make up about 50% of all phospholipids. Increased phosphatidylcholine levels have been described in vitro in the context of brome mosaic virus replication as well as after infections with dengue virus, polio virus, or hepatitis C virus [15]. Brome mosaic virus can interact with phospholipid *N*-methyltransferase choline requiring protein 2 (Cho2p), an enzyme which catalyzes the conversion of phosphatidylethanolamine to phosphatidylcholines. This interaction induces activity of this enzyme and leads to phosphatidylcholine increase in the infected cell [15,16]. This mechanism suggests that an accumulation of phosphatidylcholines in virus infected tissue may not be mediated via immune cells but directly by the interaction between host cell and virus, for instance by viral interference in biosynthetic cell processes. While it remains to be proven that this process also exists in the human CNS, it is tempting to speculate that the increased concentration of PC.ae.C36.3 detected in the CSF samples from patients without pleocytosis results from direct interactions between enterovirus and meningeal cells. PC.ae.C36.3 belongs to a subgroup of unsaturated phosphatidylcholines characterized by an ether linkage to one alkyl chain and one polyunsaturated fatty acid. Different functions have been described for this subtype of phosphatidylcholine, such as roles in lipid signal pathways and in cytoprotection under oxidative stress. Zhang et al. observed decreased concentrations of this phosphatidylcholine in plasma and synovial fluid of patients with osteoarthritis who also suffer from diabetes mellitus, suggesting that enhanced lipid peroxidation could play a role in these patients [17]. A specific role of phosphatidylcholines as protectors against cytotoxic effects in human diseases has not been proven. In our previous study we found that both PC.ae.C36.2 and PC.ae.C36.3 are also elevated in CSF samples from patients with VZV meningitis or encephalitis, but that they are not among the most accurate biomarkers for this CNS infection, which has a potentially more severe clinical course than enteroviral meningitis [12]. It is therefore possible that increased concentrations (as seen in our subgroup without pleocytosis) may reflect aspects of virus-host cell interactions that are relatively independent of the viral species and result from non-specific cellular responses in the sense of an intrinsic immune response. 

Measurements of PC.ae.C36.2 concentrations in septic shock, breast cancer, or Huntington’s disease in peripheral blood samples did not reveal any significant concentration changes [18,19,20]. Thus, future studies should address the question of whether altered concentrations of this metabolite are preferentially found in CNS disorders. 

Having shown that PC.ae.36.3 is a highly accurate biomarker for enteroviral meningitis with normal CSF cell count, the question arises whether it is feasible to translate this finding into clinical applications, for instance as a rapid diagnostic tool. Fluorometric assays can be used to quantify total phosphatidylcholine levels and are offered in routine laboratories. However, analyzing a single sample is expensive and time-consuming, so samples are often collected over a period of time and then analyzed in batch. However, this is not feasible for an acute situation like meningitis. The development of simple point-of-care kits, such as a lateral flow assay for CSF [21,22], would therefore be desirable. Detection and quantification of PC.ae.C36.3 with such an assay could be a helpful tool in clinical practice to identify enteroviral meningitis patients in whom standard CSF parameters suggest the absence of an infectious process. A positive result would trigger further investigations including PCR for enteroviruses. Further investigations on specificity, sensitivity, and cut-off values (which could not be obtained in the present study due to the small group sizes) are required in order to implement such a measurement in routine clinical diagnostic workflows.

As opposed to the subgroup with normal cell count, regulation of amino acid and biogenic amines metabolism was apparent in the pleocytosis subgroup, and an amino acid “signature” persisted in the combined group also containing the non-pleocytosis sample, suggesting that alterations in the associated pathways (albeit milder) also occur in the absence of CSF pleocytosis. Asparagine plays an important role in cell proliferation. Inside cells it controls uptake of certain amino acids whose levels are critical for activity of the mTOR complex, a promotor of proliferation [23]. 

Glycine is an inhibitory neurotransmitter that regulates several neurological functions [24,25]. Furthermore, it has been described to have immunomodulatory or cytoprotective functions and to play a role in inflammation during trauma or sepsis [25]. In our previous analysis of CSF metabolites in VZV reactivation, we identified glycine as one of the most highly elevated metabolites in CSF from patients with VZV meningoencephalitis, suggesting that there could be a connection between elevated glycine levels and stress in parenchymal cells during viral CNS infection [12]. Kynurenine was detected >LOD exclusively in meningitis with pleocytosis. It is a breakdown product of tryptophan and increased kynurenine concentrations in CSF are most likely due to induction of the tryptophan-kynurenine pathway in inflammatory cells [26]. Our results suggest that this pathway is also activated in enteroviral meningitis characterized by neuroinflammation that is pronounced enough to induce pleocytosis. Further work is necessary to assess the pathophysiologic significance of this finding.

Our study is limited by the small number of patients, especially in the enterovirus subgroups. In order to gain robust data even with low numbers of participants, we performed an internal validation. Thus, it was still possible to identify several potential biomarkers. These require validation in a larger group of patients, including calculation of sensitivity and specificity, which was not possible with our data. Furthermore, the control group was not formed by healthy patients, and CSF findings in this group may therefore not entirely reflect findings expected in healthy individuals from the general population. However, lumbar puncture in healthy individuals is ethically disputable. We thus used CSF obtained during routine diagnostic evaluations from patients where, in the final diagnosis, inflammation of the nervous system was excluded. Finally, our mass-spectrometric assay allowed detection of only a portion of all metabolites that are found in the CSF, and it is therefore possible that we did not capture the full spectrum of metabolite biomarkers.

Taken together, our results suggest: (1) that metabolite profiles in CSF of patients with enteroviral meningitis differ considerably from patients without infectious CNS disease, and (2) that this high degree of metabolite “reprogramming” reveals accurate CSF biomarkers that can aid in the diagnosis of enteroviral meningitis, even in the clinically challenging subgroup of patients with a clinical suspicion of meningitis but normal CSF cell count. 

## 4. Materials and Methods 

### 4.1. Study Population

CSF samples were obtained during routine lumbar puncture and collected prospectively as part of establishment of a CSF biobank encompassing a variety of neurological disorders. This study was approved by the Ethics Committee of Hannover Medical School (file no. 7385-21.03.2017; 21 March 2017) and was conducted according to the Helsinki Declaration. Inclusion criteria for enteroviral meningitis (*n =* 10, abbreviated as EntM (all)) were clinical suspicion of meningitis and detection of enteroviruses in CSF by polymerase chain reaction (PCR). This group could be subdivided into 50% (*n =* 5) with normal CSF cell count (0–4 cells/μL; EntM (0–4)) and 50% (*n =* 5) with elevated cell count (≥5 cells/μL; EntM (≥5)). The non-inflamed control group (*n =* 19) consisted of patients with normal CSF cell count (0–4 cells/μL) and a clinical diagnosis of (1) idiopathic facial paresis without evidence of an infectious etiology (Bell’s palsy, *n =* 9) and (2) normal pressure hydrocephalus (NPH, *n =* 10), defined as normal CSF pressure, cranial computed tomography, or magnetic resonance tomography indicative of NPH in combination with at least one symptom of the Hakim triad [27]. After lumbar puncture, the following routine CSF parameters were determined within 2 h: cell count (counted manually with a Fuchs-Rosenthal counting chamber), lactate concentration, protein concentration (Bradford dye-binding assay), Q-albumin ratio (calculated from albumin concentration in CSF/albumin concentration in serum), and IgG-index (IgG concentration in CSF/IgG concentration in serum divided by Q-albumin ratio). Albumin and IgG concentrations were measured with a latex-enhanced assay by kinetic nephelometry (Beckman Coulter IMMAGE). Quality of all methods is assured by external quality control programs (CSF survey of INSTAND).

### 4.2. Metabolite Profiling

CSF metabolite concentrations were measured with the AbsoluteIDQ™-p180 kit (Biocrates Life Sciences, Innsbruck, Austria), using a combination of liquid chromatography tandem mass spectrometry (LC-MS/MS) and direct flow injection analysis MS/MS, as described by Kuhn et al., 2018 [12]. With this system, it is possible to detect up to 188 analytes, comprising 21 amino acids, 21 biogenic amines, 91 glycerophospholipids (phosphatidyl- and lysophosphatidylcholines), 40 acylcarnitines, 15 sphingolipids (sphingo- and hydroxysphingomyelins), and the sum of hexoses. The following phosphatidylcholine (PC) nomenclature is used: aa: both side chains are fatty acids linked to a glycerol backbone by ester bonds, ae: one of those is a fatty alcohol linked to a glycerol backbone by another bond; and C*x*:*y*: *x* = total number of carbon atoms and *y* = total number of double bonds in both fatty acid chains. We used the Analyst^®^ (version 1.5.2, Sciex Framingham, MA, USA) and MetIDQ™ software (Biocrates) for peak integration and calculation of metabolite concentrations. A two-step quality screen was applied to select the subgroup of detectable analytes to be included in the present analysis. First, all analytes that were detected >limit of detection (LOD) in ≥75% of all samples were selected. Values <LOD were then replaced by k-nearest-neighbor imputation, as described in the R package “Biocomb” [28]. Second, those analytes which did not pass the first step because of values <LOD in the control group (and which might therefore constitute biomarkers for enteroviral meningitis) were identified and included in the analysis, but without imputing values <LOD.

### 4.3. Statistical Analyses

Significance of between- and across-group comparisons was determined with the Mann-Whitney U and Kruskall-Wallis tests, respectively, defining significance as *p* <0.05 unless stated otherwise. Discriminatory ability of the metabolite biomarkers was assessed by receiver operating characteristic (ROC) curve analysis, defining significant areas under the curve (AUC) by asymptotic *p* values of <0.05 and lower bound confidence intervals (CI) of >0.5. The group with the higher CSF cell count constituted the enumerator for calculating ratios of mean concentrations (“fold change”), and the positive state in ROC analysis. Due to the small sample size and lack of an external validation cohort, an internal cross validation was performed via the leave-one-out (jackknife) procedure. In this method, the cohort is divided into a training subset (total *n*–1) and a validation subset (*n =* 1). The best classifier on the training subset is computed and tested on the validation subset in an iterative process, where each sample is used as the validation subset. The classifiers are then ranked in order of selection frequency. To select optimal biomarker combinations, we constructed a random forest classifier based on the biomarker candidates with the highest AUC values and tested the classifier on the single sample that was left out during the corresponding iteration of the jackknife method. The most frequently selected classifiers were then re-evaluated by the jackknife method once again. To reduce selection bias, we repeated the procedure with 1000 bootstrap samples in order to compute a confidence interval for the AUC of the final classifier.

## 5. Conclusions

Our results demonstrate: (1) that despite its relatively mild clinical manifestations, there is a considerable degree of CSF metabolite reprogramming in enteroviral meningitis, and (2) that CSF metabolite analysis can be a helpful tool in establishing a differential diagnosis in clinically suspected enterovirus meningitis. Further validation of PC.a.e.36.3. as a potential biomarker and establishing easy and quick detection assays might enable its use as a point-of-care test (for instance in the emergency room or at the bedside) to trigger further investigations, including PCR for enteroviruses.

## Figures and Tables

**Table 1:** Demographic features and diagnostic blood and CSF parameters.

	Control	EntM					
		Subgroup			*p* Value ^§^, Control vs.		
	(*n =* 19)	All (*n =* 10)	0–4 Cells (*n =* 5)	≥5 Cells (*n =* 5)	EntM All	EntM 0–4 Cells	EntM ≥5 Cells
**Age (Years)**							
Median	48 (22–77)	32.5 (22–76)	47 (33–76)	30 (22–32)	0.22	0.52	0.01
Mean (SD)	48 (16.1)	41 (19.4)	55 (19)	27.8 (4.5)			
**Sex**							
Female	58% (11)	40% (4)	40% (2)	40% (2)	0.37	0.48	0.48
Male	42% (8)	60% (6)	60% (3)	60% (3)			
**Blood** **Parameter**							
**Leukocytes (1000/µL) (normal range 3.6–10.5)**							
Median	7.4 (4.6–11.9)	7 (4–14)	6.6 (5.1–9.8)	7.3 (4–14)	0.55	0.5	0.80
Mean (SD)	7.8 (2.3)	7.4 (2.9)	7.1 (1.83)	7.7 (3.8)			
**CRP (mg/L) (normal range 0–5)**							
Median	3 (1–31)	4 (1–39)	4 (1–17)	4 (1–39)	0.09	0.18	0.20
Mean (SD)	5.4 (8.3)	9.5 (11.6)	7.4 (6.5)	11.6 (15.8)			
**CSF Parameter**							
**Cell count (1/µL) (normal range 0–4)**							
Median	1.3 (0.3–4)	9.2 (0.7–619)	1.7 (0.7–4)	97.3 (14.3–619)	0.008	n/a	n/a
Mean (SD)	1.6 (1.1)	129.1 (235.3)	2 (1.35)	256.2 (290.3)			
**Protein *(*g/L) (0.17–0.52)**							
Median	0.39 (0.26–0.83)	0.51 (0.24–0.98)	0.52 (0.24–0.98)	0.54 (0.46–0.75)	0.14	0.41	0.15
Mean (SD)	0.45 (0.15)	0.57 (0.19)	0.58 (0.51)	0.56 (0.16)			
**Lactate *(*mmol/L) (normal range 1.1–1.9)**							
Median	1.57 (1.2–2.1)	1.88 (1.55–3.55)	1.7 (1.55–2.18)	2.15 (1.84–3.55)	0.007	0.24	0.003
Mean (SD)	1.6 (0.25)	2.1 (0.62)	1.76 (0.5)	2.43 (0.71)			
**IgG Index**							
Median	0.49 (0.43–0.6)	0.54 (0.47–0.63)	0.55 (0.49–0.63)	0.52 (0.47–0.54)	0.041	0.035	0.28
Mean (SD)	0.5 (0.05)	0.53 (0.05)	0.56 (0.05)	0.51 (0.03)			
**Blood-CSF-Barrier Dysfunction**							
No	58% (11)	20% (2)	40% (2)	0	0.037	0.31	0.023
Light	42% (8)	70% (7)	40% (2)	100% (5)			
Moderate	0	10% (1)	20% (1)				
Severe	0	0	0	0			

^§^ All *p* values determined with Mann-Whitney U test except BCB dysfunction (Chi^2^ test). Abbreviations: EntM (all): enterovirus meningitis, EntM (0–4): enterovirus meningitis with CSF cell count 0–4/µL, EntM (≥5): enterovirus meningitis patient with CSF cell count ≥5/µL.

**Table 2:** Quantitative evaluation of CSF metabolite biomarkers for enteroviral meningitis.

EntM (all) vs. Control	AUC	*p*-Value	Lower CI	Ratio of Means	Selection Frequency
PC.ae.C36.2*	0.832	0.00265	0.653	1.58	1.000
Asn	0.826	0.00316	0.621	1.35	1.000
PC.ae.C36.3	0.826	0.00316	0.634	1.82	1,000
Gly	0.816	0.00445	0.601	1.67	1.000
C5.1	0.808	0.00568	0.656	1.25	0.966
PC.aa.C34.2	0.808	0.00568	0.592	1.55	1.000
PC.ae.C38.5	0.797	0.00779	0.647	1.69	0.931
H1	0.795	0.00843	0.625	0.84	0.776
Ser	0.792	0.00907	0.574	0.75	0.707
PC.aa.C34.3	0.776	0.01406	0.587	1.43	0.397
**Ent M (0-4 cells) vs. Control**					
PC.ae.C36.3*	0.874	0.00816	0.590	0.46	1.000
C2	0.821	0.02675	0.548	0.44	1.000
PC.ae.C36.5	0.821	0.02675	0.556	0.60	0.958
C0	0.811	0.03287	0.530	0.69	1.000
PC.aa.C36.5	0.811	0.03287	0.593	0.57	0.958
C5.1	0.805	0.03618	0.565	0.79	0.917
**Ent M (≥5 cells) vs. Control**					
Kyn*	1.000	2.35 × 10^−5^	1.000	6.39	1.000
Ser	0.989	7.06 × 10^−5^	0.937	0.56	1.000
Asn	0.968	0.00026	0.872	1.53	1.000
Gly	0.926	0.00167	0.748	1.73	1.000
Lys	0.916	0.00240	0.792	0.66	0.958
PC.ae.C36.2	0.895	0.00461	0.738	1.54	0.958
PC.aa.C34.2	0.884	0.00619	0.710	1.61	1.000
Tyr	0.884	0.00619	0.731	0.65	0.925
Leu	0.879	0.00706	0.654	0.65	0.883
H1	0.853	0.01360	0.669	0.82	0.467
**EntM (≥5 cells) vs. EntM (0–4 cells)**					
Kyn*	1.000	0.00397	1.000	6.39	1.000
Ser	1.000	0.00397	1.000	0.59	1.000
Leu	0.920	0.02381	0.700	0.58	0.940
Tyr	0.920	0.02381	0.667	0.68	0.900
PC.aa.C32.0	0.920	0.02381	0.714	0.76	0.940
PC.aa.C34.1	0.920	0.02381	0.672	0.71	0.940
Phe	0.880	0.04365	0.577	0.77	0.333
Val	0.880	0.04365	0.583	0.76	0.407
Creatinine	0.880	0.04365	0.577	0.77	0.367
PC.ae.C36.5	0.880	0.04365	0.577	0.57	0.433

The table lists all significant (CI > 0.5, *p* < 0.05) biomarkers, up to a maximum of 10, for each 2-group comparison. AUC, area under the ROC curve; *p* value, asymptotic *p* value of the ROC curve; CI, lower CI of the ROC curve. Ratio of mean concentrations (“fold change”), the more inflamed group (named first in column 1) constituting the enumerator. Selection frequency was determined by leave-one-out cross-validation (1.0 = always selected, i.e., most robust biomarker; 0.0 = never selected, i.e. least robust biomarker). * Most robust biomarker on basis of highest selection frequency, greatest AUC, and lowest *p* value.

**Table 3:** Comparison of standard CSF parameters and metabolite biomarkers.

	Controls vs.		
	EntM (All)	EntM (0–4 Cells)	EntM (≥5 Cells)
**Standard CSF Parameter**			
Leukocyte count	0.80 **	n/a	n/a
Protein concentration	0.67	0.62	0.72
IgG-index	0.73 *	0.81*	0.66
Lactate	0.81 **	0.67	0.94 **
BCB dysfunction	0.71	0.63	0.79 *
**Best Single Metabolites ^1^**			
Best internally validated marker	PC.ae.C36.2	PC.ae.C36.3	Kyn
AUC	0.83 **	0.87 **	1.0 ***
**Best Metabolite Classifier ^2^**			
No. of markers	3	3	1
Markers (frequency)	Asn (1.0) Gly (1.0) PC.ae.C36.3 (1.0)	PC.ae.C36.3 (1.0) PC.ae.C36.5 (0.96) PC.ae.C38.5 (0.6)	Kyn (1.0)
AUC (95% CI)	0.92 *** (0.61–1.0)	0.99 *** (0.53–1.0)	1.0 *** (1.0–1.0)

Values correspond to areas under the receiver operator characteristic (ROC) curve (AUC) performed on continuous variables. Asymptotic significance: * *p* ≤ 0.05, ** *p* ≤ 0.01, *** *p* ≤ 0.001. Underlined values: lower confidence interval (CI) > 0.5. ^1^ According to the frequencies of selection (0 = never selected; 1 = always selected) in the leave-one-out cross-validation listed in Table 2. The marker with the higher AUC was selected if two markers had the same frequencies. ^2^ Biomarker combination with highest discriminatory ability identified by random forest construction, as outlined in Methods. CIs of AUCs were evaluated based on 1000 bootstrap samples of the same size as the original data drawn with replacement. Only the metabolites were considered.

## Abbreviations

AUC	Area under the curve
BCB	Blood-CSF-Barrier
CI	Confidence interval
CRP	C-reactive protein
CSF	Cerebrospinal fluid
CNS	central nervous system
Cho2p	Choline requiring protein 2
ER	Emergency room
EntM	Enterovirus meningitis
HSV	Herpes simplex virus
IgG	Immunoglobulin G
LOD	Limit of detection
mTOR	Mechanistic target of rapamycin
NPH	Normal pressure hydrocephalus
PC	Phosphatidylcholine
PCR	Polymerase chain reaction
RNA	Ribonucleic acid
ROC	Receiver operating characteristic
VZV	Varicella zoster virus

## References

[1] Hoffman O., Weber R.J. (2009). Pathophysiology and treatment of bacterial meningitis. Ther. Adv. Neurol. Disord..

[2] Bartt R. (2012). Acute bacterial and viral meningitis. Continuum.

[3] De Ory F., Avellon A., Echevarria J.E., Sanchez-Seco M.P., Trallero G., Cabrerizo M., Casas I., Pozo F., Fedele G., Vicente D. (2013). Viral infections of the central nervous system in Spain: A prospective study. J. Med. Virol..

[4] Dumaidi K., Frantzidou F., Papa A., Diza E., Antoniadis A. (2006). Enterovirus meningitis in Greece from 2003–2005: Diagnosis, CSF laboratory findings, and clinical manifestations. J. Clin. Lab. Anal..

[5] Majed B., Zephir H., Pichonnier-Cassagne V., Yazdanpanah Y., Lestavel P., Valette P., Vermersch P. (2009). Lumbar punctures: Use and diagnostic efficiency in emergency medical departments. Int. J. Emerg. Med..

[6] Glimaker M., Johansson B., Grindborg O., Bottai M., Lindquist L., Sjolin J. (2015). Adult bacterial meningitis: Earlier treatment and improved outcome following guideline revision promoting prompt lumbar puncture. Clin. Infect. Dis. Off. Publ. Infect. Dis. Soc. Am..

[7] Ostergaard A.A., Sydenham T.V., Nybo M., Andersen A.B. (2017). Cerebrospinal fluid pleocytosis level as a diagnostic predictor? A cross-sectional study. BMC Clin. Pathol..

[8] Ahlbrecht J., Hillebrand L.K., Schwenkenbecher P., Ganzenmueller T., Heim A., Wurster U., Stangel M., Suhs K.W., Skripuletz T. (2018). Cerebrospinal fluid features in adults with enteroviral nervous system infection. Int. J. Infect. Dis. IJID Off. Publ. Int. Soc. Infect. Dis..

[9] Debiasi R.L., Tyler K.L. (2004). Molecular methods for diagnosis of viral encephalitis. Clin. Microbiol. Rev..

[10] Ihekwaba U.K., Kudesia G., McKendrick M.W. (2008). Clinical features of viral meningitis in adults: Significant differences in cerebrospinal fluid findings among herpes simplex virus, varicella zoster virus, and enterovirus infections. Clin. Infect. Dis. Off. Publ. Infect. Dis. Soc. Am..

[11] Patriquin G., Hatchette J., Forward K. (2012). Clinical presentation of patients with aseptic meningitis, factors influencing treatment and hospitalization, and consequences of enterovirus cerebrospinal fluid polymerase chain reaction testing. Can. J. Infect. Dis. Med Microbiol. J. Can. Des Mal. Infect. Et De La Microbiol. Med..

[12] Kuhn M., Suhs K.W., Akmatov M.K., Klawonn F., Wang J., Skripuletz T., Kaever V., Stangel M., Pessler F. (2018). Mass-spectrometric profiling of cerebrospinal fluid reveals metabolite biomarkers for CNS involvement in varicella zoster virus reactivation. J. Neuroinflamm..

[13] Hosmer D.W., Lemeshow S. (2000). Applied Logistic Regression.

[14] Demler O.V., Pencina M.J., D’Agostino R.B. (2013). Impact of correlation on predictive ability of biomarkers. Stat. Med..

[15] Zhang J., Zhang Z., Chukkapalli V., Nchoutmboube J.A., Li J., Randall G., Belov G.A., Wang X. (2016). Positive-strand RNA viruses stimulate host phosphatidylcholine synthesis at viral replication sites. Proc. Natl. Acad. Sci. USA.

[16] Boumann H.A., Chin P.T., Heck A.J., De Kruijff B., De Kroon A.I. (2004). The yeast phospholipid N-methyltransferases catalyzing the synthesis of phosphatidylcholine preferentially convert di-C16:1 substrates both in vivo and in vitro. J. Biol. Chem..

[17] Zhang W., Randell E.W., Sun G., Likhodii S., Liu M., Furey A., Zhai G. (2017). Hyperglycemia-related advanced glycation end-products is associated with the altered phosphatidylcholine metabolism in osteoarthritis patients with diabetes. PLoS ONE.

[18] Cambiaghi A., Pinto B.B., Brunelli L., Falcetta F., Aletti F., Bendjelid K., Pastorelli R., Ferrario M. (2017). Characterization of a metabolomic profile associated with responsiveness to therapy in the acute phase of septic shock. Sci. Rep..

[19] Qiu Y., Zhou B., Su M., Baxter S., Zheng X., Zhao X., Yen Y., Jia W. (2013). Mass spectrometry-based quantitative metabolomics revealed a distinct lipid profile in breast cancer patients. Int. J. Mol. Sci..

[20] Mastrokolias A., Pool R., Mina E., Hettne K.M., van Duijn E., van der Mast R.C., van Ommen G., t Hoen P.A., Prehn C., Adamski J. (2016). Integration of targeted metabolomics and transcriptomics identifies deregulation of phosphatidylcholine metabolism in Huntington’s disease peripheral blood samples. Metab. Off. J. Metab. Soc..

[21] Yetisen A.K., Akram M.S., Lowe C.R. (2013). Paper-based microfluidic point-of-care diagnostic devices. Lab Chip.

[22] Boulos A., Fairley D., McKenna J., Coyle P. (2017). Evaluation of a rapid antigen test for detection of Streptococcus pneumoniae in cerebrospinal fluid. J. Clin. Pathol..

[23] Krall A.S., Xu S., Graeber T.G., Braas D., Christofk H.R. (2016). Asparagine promotes cancer cell proliferation through use as an amino acid exchange factor. Nat. Commun..

[24] Perez-Torres I., Zuniga-Munoz A.M., Guarner-Lans V. (2017). Beneficial Effects of the Amino Acid Glycine. Mini Rev. Med. Chem..

[25] Lopez-Corcuera B., Geerlings A., Aragon C. (2001). Glycine neurotransmitter transporters: An update. Mol. Membr. Biol..

[26] Badawy A.A. (2017). Kynurenine Pathway of Tryptophan Metabolism: Regulatory and Functional Aspects. Int. J. Tryptophan Res..

[27] Adams R.D., Fisher C.M., Hakim S., Ojemann R.G., Sweet W.H. (1965). Symptomatic Occult Hydrocephalus with “Normal” Cerebrospinal-Fluid Pressure. A Treatable Syndrome. N. Engl. J. Med..

[28] Novoselova N., Wang J., Pessler F., Klawonn F. Biocomb: Feature Selection and Classification with the Embedded Validation Procedures for Biomedical Data Analysis. R Package Version 0.4. https://CRAN.R-project.org/package=Biocomb.

